# Single-Molecule Analysis Reveals the Kinetics and Physiological Relevance of MutL-ssDNA Binding

**DOI:** 10.1371/journal.pone.0015496

**Published:** 2010-11-12

**Authors:** Jonghyun Park, Yongmoon Jeon, Daekil In, Richard Fishel, Changill Ban, Jong-Bong Lee

**Affiliations:** 1 Department of Physics, Pohang University of Science and Technology (POSTECH), Pohang, Kyungbuk, Republic of Korea; 2 Department of Molecular Virology, Immunology and Medical Genetics, The Ohio State University Medical Center, Columbus, Ohio, United States of America; 3 Physics Department, The Ohio State University, Columbus, Ohio, United States of America; 4 Department of Chemistry, Pohang University of Science and Technology (POSTECH), Pohang, Kyungbuk, Republic of Korea; 5 School of Interdisciplinary Bioscience and Bioengineering, Pohang University of Science and Technology (POSTECH), Pohang, Kyungbuk, Republic of Korea; Indiana University School of Medicine, United States of America

## Abstract

DNA binding by MutL homologs (MLH/PMS) during mismatch repair (MMR) has been considered based on biochemical and genetic studies. Bulk studies with MutL and its yeast homologs Mlh1-Pms1 have suggested an integral role for a single-stranded DNA (ssDNA) binding activity during MMR. We have developed single-molecule Förster resonance energy transfer (smFRET) and a single-molecule DNA flow-extension assays to examine MutL interaction with ssDNA in real time. The smFRET assay allowed us to observe MutL-ssDNA association and dissociation. We determined that MutL-ssDNA binding required ATP and was the greatest at ionic strength below 25 mM (*K_D_* = 29 nM) while it dramatically decreases above 100 mM (*K_D_*>2 µM). Single-molecule DNA flow-extension analysis suggests that multiple MutL proteins may bind ssDNA at low ionic strength but this activity does not enhance stability at elevated ionic strengths. These studies are consistent with the conclusion that a stable MutL-ssDNA interaction is unlikely to occur at physiological salt eliminating a number of MMR models. However, the activity may infer some related dynamic DNA transaction process during MMR.

## Introduction

MutL homologs (MLH/PMS) are key components of mismatch repair (MMR). Mismatch recognition by MutS homologs (MSH) results in long-lived ATP-bound sliding clamps that recruit MLH/PMS, which in turn stimulate the DNA transaction activities of several downstream effectors. In *Escherichia coli* (*E. coli*), these downstream effectors include MutH and UvrD. For example, the *E. coli* MutL stimulates the MutH endonuclease activity on a hemimethylated d(GATC) that directs excision repair to the newly replicated strand as well as enhances the UvrD helicase activity required for the strand excision process [1], [2].

MutL has been suggested to bind ssDNA in the presence of ATP; an activity that may play an important role in its interaction with downstream effectors such as UvrD [3], [4], [5]. Biochemical and structural studies suggest that the C-terminal region of *E. coli* MutL forms a stable homodimer (LC20) [6], [7] while the N-terminal domain (LN40) contains a GHKL ATPase site [8] that dimerizes upon binding to ATP [9], [10]. Together, the resulting structure of the ATP-bound MutL appears to form a cavity via a flexible linker that contains a positively charged cleft [9]. This ATP-dependent MLH/PMS conformational change appears to be modulated by the ATP binding and hydrolysis cycle even in the absence of DNA [11].

It is the positively charged cavity formed by ATP-bound MutL that appears to contain the ssDNA-binding domain [9], [11]. However, the properties and roles of MutL-ssDNA binding in MMR are poorly understood. Studies that appear to support a role for MutL-ssDNA binding in MMR include: 1) MutL appears to bind unmethylated ssDNA better than methylated ssDNA or unmethylated/methylated dsDNA [3], 2) ssDNA stimulates the MutL ATPase [9], and 3) the MutL(R266E) mutant protein that displays a weak ssDNA-binding affinity and that lacks ssDNA-stimulated ATPase activity genetically behaves like a *mutL* null mutant [5]. While these studies appear to correlate MutL-ssDNA binding with MMR, all of the ssDNA binding studies *in vitro* were performed at non-physiological ionic strengths [6], [12]. Moreover, there are reports that suggest MutL does not bind to DNA at physiological salt and that DNA binding is not required for MMR [13], [14].

We have developed single-molecule assays that examine the lengthening of random-coiled ssDNA, which results from MutL binding. These assays, single-molecule Förster Resonance Energy Transfer (smFRET) [15] and a single molecule flow-extension assay [16], [17], have allowed us to study the kinetics of the MutL-ssDNA interaction in real time. Our studies examined the interaction between MutL and ssDNA in the absence of other MMR proteins. Together the single-molecule analysis detailed both the heterogeneity and the physiological relevance of the MutL-ssDNA interaction.

## Results

### *E. coli* MutL binds and stretches ssDNA

**Figure 1:**
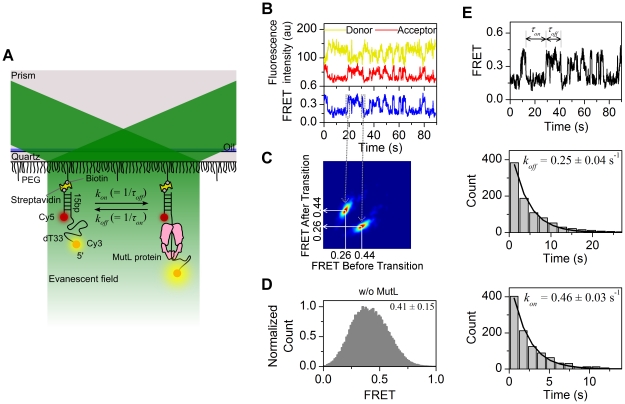
Single-molecule FRET analysis of MutL-ssDNA binding. (**A**) Schematic representation of a single-molecule FRET system. The Evanescent field resulting from Total Internal Reflection excites a Cy3 donor fluorophore that may FRET with a Cy5 acceptor fluorophore. A partial duplex DNA attached to the PEG-biotin surface via a biotin-streptavidin linker contains a 5′-dT33 single-stranded tail labeled with the donor Cy3 and an acceptor Cy5 linked to the dsDNA/ssDNA junction. The distance between fluorophores changes with MutL-ssDNA binding affecting the FRET efficiency. (**B**) Representative single molecule trace of the Cy3 and Cy5 fluorescence intensity (top panel) and the resulting FRET (bottom panel) with 50 nM MutL in 25 mM NaCl. The binding of MutL reduces the FRET. (**C**) A transition density plot in the presence of MutL. There are two FRET states of 0.44 and 0.26 FRET values, respectively. The plot consists of 2,362 transitions from 257 traces. (**D**) Histogram of FRET values from populations of single molecules normalized to the peak count. The FRET efficiency in the absence of MutL is 0.41±0.15 (mean ± s.d.; n = 74,634 points). (**E**) Representative trace in the presence of 50 nM MutL in 25 mM NaCl. The FRET efficiency for *τ_on_* and *τ_off_* represent the association and dissociation time of MutL, respectively (upper panel). Rate constants, *k_on_* = 1/*τ_off_* and *k_off_* = 1/*τ_on_* were determined by fitting an exponential decay function to the histogram derived from a population of dwell times (lower panels).

The ssDNA binding activity of MutL was examined using a partial duplex DNA that consisted of 15 bp double-stranded DNA (dsDNA) with a 33-deoxythymidine nucleotide (dT33) 5′-overhang. An acceptor Cy5 at the ssDNA/dsDNA junction and a donor Cy3 on the end of the 5′-overhang were used as FRET pairs. A 5′-biotin anchored the DNA substrate to the quartz slide glass coated with PEG-biotin using a streptavidin linker (Fig. 1A). Injection of MutL (50 nM) in 25 mM NaCl resulted in the abrupt decrease of the acceptor signal at 3 s that was maintained for 13 s (Fig. 1B). This pattern and the anticorrelation signals between a donor and an acceptor were repetitive for 85 s (Fig. 1B).

To identify the resulting FRET states, we applied hidden Markov modeling (HMM) analysis, which determines the states with a distinct FRET efficiency (Data Analysis in Materials and Methods). The HMM analysis discerned two FRET states resulting from the ssDNA binding by MutL, which was presented in a transition density plot (Fig. 1C) [18]. The transition density plot represents the transition distribution between two distinct FRET states from 0.44 to 0.26 and from 0.26 to 0.44 along each axis (FRET before transition to FRET after transition). It indicates that the random coiled ssDNA tail that was not bound by MutL displayed a constant FRET efficiency of 0.44. However, the lower FRET efficiency of 0.26 appears to be the result of ssDNA binding by MutL. The lower FRET value was cycled with the FRET efficiency observed in the absence of MutL (0.41±0.15; mean ± s.d.; Fig. 1D). These results indicate a time-dependent distance increased between the donor and the acceptor when MutL bound to ssDNA; providing a convenient assay for mutational and kinetic analysis (Fig. 1A). The association (*τ_on_*) and dissociation (*τ_off_*) dwell time of FRET states may be garnered by examining individual traces of FRET efficiency (Fig. 1E). A histogram derived from a population of dwell times was fitted to a single exponential and resulted in the off-rate (*k_off_* = 1/*τ_on_* = 0.25±0.04 s^−1^, mean ± s.e.m.) and on-rate (*k_on_* = 1/*τ_off_* = 0.46±0.03 s^−1^, mean ± s.e.m.) for 50 nM MutL in 25 mM NaCl (Fig. 1E). The single exponential property of the dwell-time distribution indicates that the kinetics of MutL-ssDNA binding can be described by a single rate constant.

**Figure 2:**
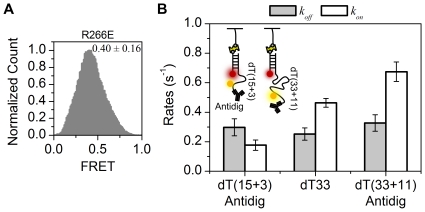
The effect of ssDNA-tail with different lengths and blocked ssDNA-ends on the binding by MutL. (**A**) Histogram of FRET values normalized to the peak count. The FRET efficiency in the presence of MutL(R266E) is 0.40±0.16 (mean ± s.d.; n = 91,386 points). (**B**) Schematic representation of the end-blocked ssDNAs (inset) and the kinetic rates (*k_on_*, *k_off_*) of the end-blocked 18 nt ssDNA [dT(15+3)] (n = 251 traces), the open-ended 33 nt ssDNA dT33 (n = 257 traces), and the end-blocked 44 nt ssDNA [dT(33+11)] (n = 168 traces).

To assess whether the FRET change is due to the specific ssDNA binding by MutL, we performed smFRET studies with the MutL(R266E) mutant protein. The mutant protein substitutes a negatively charged glutamic acid for a positively charged arginine residue within the opening formed by the linked LC20 and LN40 peptides that contains the putative DNA binding region [9], [19]. We found that the FRET efficiency of MutL(R266E) was 0.40±0.16 (mean ± s.d.), which was nearly identical to that observed in the absence of MutL (Fig. 2A). These results are consistent with the conclusion that binding by MutL stretches the ssDNA and that the MutL(R266E) is defective in this process. These studies suggest that the MutL(R266) residue plays a role in ssDNA binding. We confirmed that the length change of the ssDNA was generated by a specific MutL-ssDNA interaction since we observed no change in FRET values in the presence of UvrD helicase that moves along ssDNA from 3′ to 5′ unidirectionally to unwind duplex DNA (data not shown).

To further explore the specificity of MutL-ssDNA binding, we investigated the kinetic rate dependence on the ssDNA length. We constructed a partial duplex DNA (15 bp dsDNA) with a 44-deoxythymidine nucleotide [dT(33+11)] 5′-overhang that bears digoxigenin. The donor Cy3 was attached to the 11^th^ dT from the 5′ end, which maintains the distance between Cy3 and Cy5 similar to the dT33 substrate (Fig. 2B inset). The 5′ end of the ssDNA was blocked by anti-digoxigenin antibody, which can prevent MutL from binding to the ssDNA end and from dissociating from the end (Fig. 2B cartoon). We also prepared the partial duplex with an end-blocked 18 nt ssDNA tail. Cy3 was conjugated to a 3^rd^ nucleotide from the end of 18 nt ssDNA [dT(15+3)] (Fig. 2B cartoon). We found that the on-rate (*k_on_*) of the end-blocked dT(33+11) (0.67±0.07 s^−1^) was greater than the unblocked dT33 (0.46±0.03 s^−1^) and the end-blocked dT(15+3) (0.18±0.04 s^−1^) (Fig. 2B). This result suggests that a longer ssDNA tail increases the rate of MutL association with the ssDNA. In contrast, the off-rates (*k_off_*) of the end-blocked dT(33+11) (0.33±0.06 s^−1^), the end-free dT33 (0.25±0.04 s^−1^), and the end-blocked dT(15+3) (0.30±0.06 s^−1^) DNA substrates were not significantly different (Fig. 2B). The errors in the kinetic rates represent s.e.m. Together these results clearly suggest that the change in FRET values results from MutL-ssDNA binding. Moreover, MutL binding does not require a ssDNA end for binding; although interaction with the ssDNA/dsDNA junction can not be ruled out with the smFRET studies.

### ATP dependence of MutL binding to ssDNA

**Figure 3:**
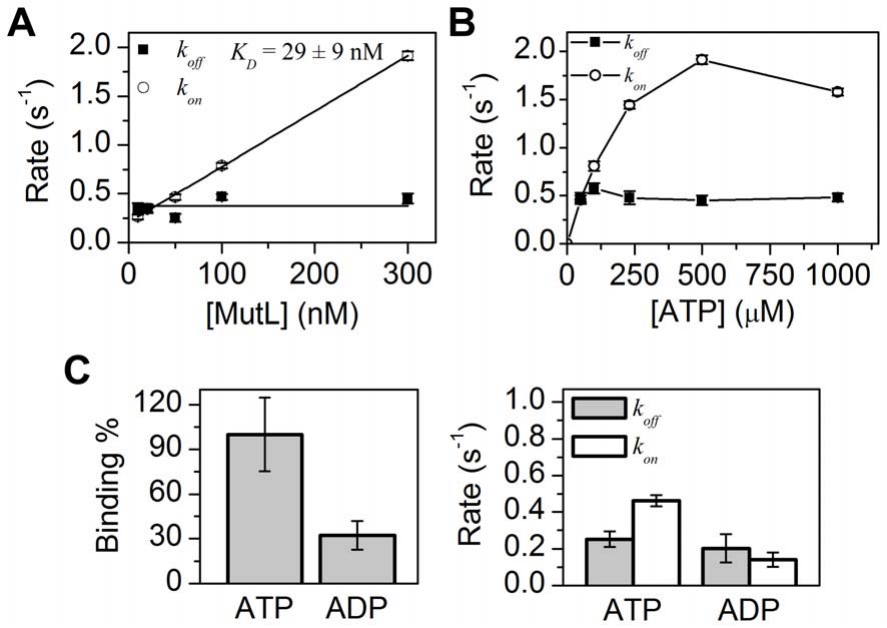
Kinetics of MutL-ssDNA binding. (**A**) MutL concentration dependence of *k_on_* and *k_off_*. The dissociation equilibrium constant in 25 mM NaCl was calculated from the intercept where *k_on_* = *k_off_* (*K_D_* = 29.9±9 nM). All the error bars represent s.e.m. [MutL] = 10 nM (n = 429 traces), 20 nM (n = 221 traces), 50 nM (n = 257 traces), 100 nM (n = 278 traces), and 300 nM (n = 455 traces). (**B**) The effect of ATP concentration on *k_on_* and *k_off_*. The *k_on_* appears to saturate at 500 µM ATP while the *k_off_* does not change significantly. All the error bars represent s.e.m. [ATP] = 50 µM (n = 223 traces), 100 µM (n = 143 traces), 230 µM (n = 235 traces), 500 µM (n = 455 traces), and 1000 µM (n = 206 traces). (**C**) The number of events and the kinetic rates (*k_on_*, *k_off_*) in the presence of ATP (n = 339 traces) and ADP (n = 166 traces).

We further characterized the kinetics of MutL-ssDNA binding (Fig. 1E). The *k_on_* was found to be proportional to the concentration of MutL, while the *k_off_* was independent of MutL concentration (Fig. 3A). We determined the dissociation constant (*K_D_*) as the intercept of *k_on_* and *k_off_* from a titration of MutL in 25 mM NaCl (*K_D_* = 29±9 nM, mean ± s.e.m.; Fig. 3A) [18].

ATP processing by MutL is essential for interactions with MutH and UvrD [4], [13]. In the absence of ATP we did not observe any significant changes in FRET efficiency in the presence of MutL. Moreover, the *k_off_* did not vary with ATP concentration. However, the *k_on_* increases with increasing ATP concentration and saturated at ∼500 µM ATP (Fig. 3B). To investigate the dependence of nucleotide for MutL-ssDNA binding, we also performed smFRET studies in the presence of ADP. We observed approximately 3-fold more MutL-ssDNA binding events in the presence of ATP compared to ADP (Fig. 3C). The *k_on_* in the presence of ADP also decreased significantly (0.14±0.04 s^−1^), while the *k_off_* (0.25±0.04 s^−1^) in the presence of ATP was not significantly different from ADP (0.20±0.08 s^−1^) (Fig. 3C). In addition, smFRET studies with the MutL(D58A) substitution mutation that does not bind ATP [5], displays no significant changes in FRET efficiency (Figure S1). These results suggest that ssDNA binding requires MutL ATP/ADP binding functions, although ADP is clearly less effective than ATP as an allosteric effector [11].

### ssDNA binding by MutL is absent at physiological ionic strength

**Figure 4:**
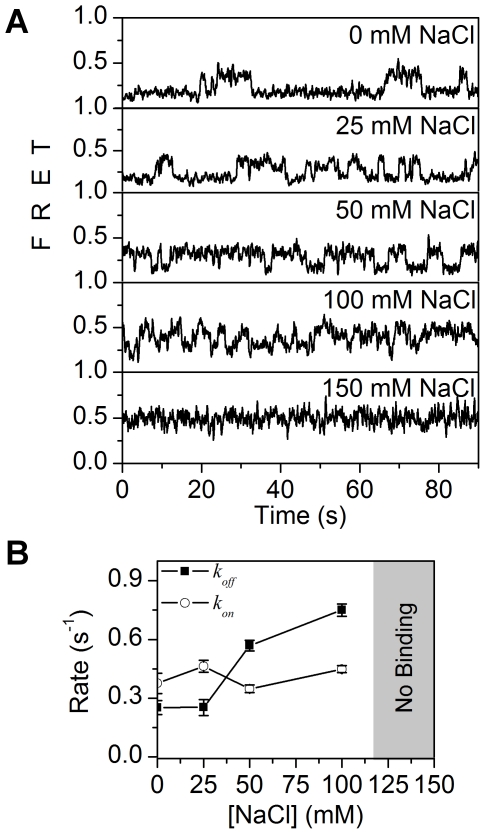
The effect of ionic strength on MutL-ssDNA binding. (**A**) Representative FRET traces at 0, 25, 50, 100, and 150 mM of NaCl. (**B**) The *k_on_* and *k_off_* at 50 nM of MutL plotted against salt concentration. We observed no significant FRET due to MutL-ssDNA binding above 100 mM NaCl. [NaCl] = 0 mM (n = 130 traces), 25 mM (n = 257 traces), 50 mM (n = 185 traces), and 100 mM (n = 278 traces).

Ionic contacts play an important role in DNA-protein interaction since negatively charged DNA phosphates specifically contact positively charged peptide residues within binding sites [20]. We examined the ionic-dependence of MutL-ssDNA binding using the smFRET system. The *τ_on_* (1/*k_off_*) dwell time of the reduced FRET efficiency induced by MutL binding decreased with the increasing salt concentration and was completely absent above 100 mM NaCl (Fig. 4A). The calculated *k_off_* increased more than 3-fold from 25 to 100 mM NaCl (Fig. 4B). In contrast, the *k_on_* was not significantly affected by similar salt concentrations (Fig. 4B). Furthermore, at 110 mM NaCl the change in FRET signals was exceedingly inaccurate and any altered FRET efficiency induced by MutL binding dramatically disappeared at 120 mM NaCl (Fig. 4B). Since the dwell time of MutL (50 nM) at ionic strengths above 100 mM is <50 ms (*k_off_*>20 s^−1^) while the *k_on_* appears relatively constant (0.41 s^−1^), we estimate the *K_D_* to be greater that 2 µM at physiological ionic strength. These results are consistent with the conclusion that MutL does not bind ssDNA at physiological ionic strength, which is coincident with previous reports [13].

### Flow-extension single-molecule analysis of MutL-ssDNA binding

**Figure 5:**
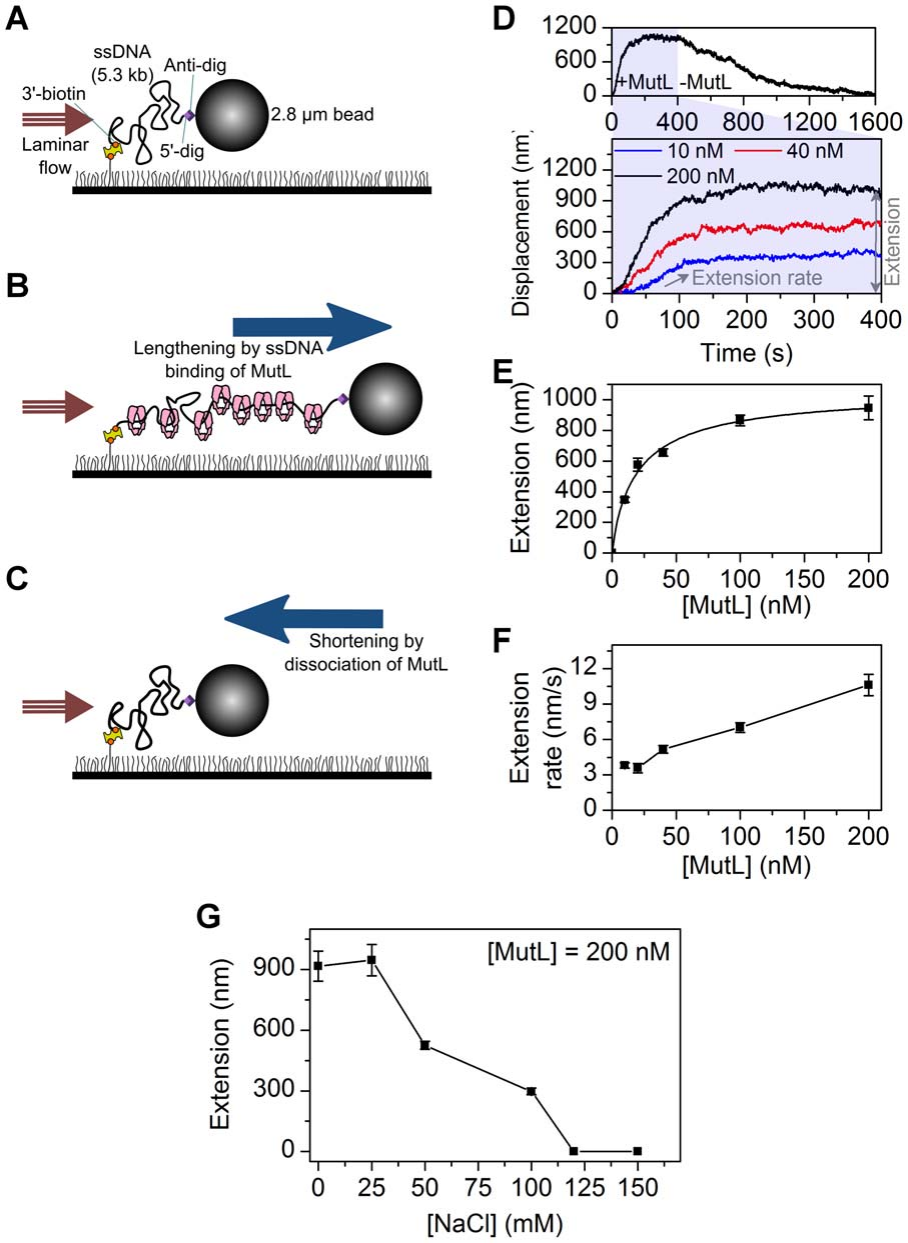
Single stranded DNA extension analysis of MutL binding activity. (**A**) A 5.3 kb ssDNA is coiled randomly at a stretching force of 2.5 pN. (**B**) MutL binding extends the ssDNA and the position of the magnetic bead at the constant force of 2.5 pN. (**C**) Washing out free MutL results in gradual dissociation of the MutL bound to the ssDNA, resulting in the shortening of magnetic bead position. (**D**) Extension vs. time at different MutL concentrations (10, 40, and 200 nM). At 400 s, the free MutL was washed out of the flow chamber and the decrease in extension representing MutL dissociation monitored at 2.5 pN force. (**E**) Representative traces and the plot of extension (amplitude) measured at increasing concentrations of MutL. [MutL] = 10 nM (n = 51 beads), 20 nM (n = 15 beads), 40 nM (n = 52 beads), 100 nM (n = 37 beads), and 200 nM (n = 20 beads). (**F**) The extension rates measured at increasing concentrations of MutL. Studies in panels D–G were performed in the presence of 500 µM ATP in 25 mM NaCl. (**G**) The extension versus salt concentrations of 0 mM (n = 20 beads), 25 mM (n = 19 beads), 50 mM (n = 27 beads), 100 mM (n = 91 beads), 120 mM, and 150 mM NaCl in the presence of 200 nM MutL and 500 µM ATP. All the error bars represent s.e.m..

It is possible that an interaction among multiple MutL proteins could alter and/or stabilize a ssDNA binding activity [12]. We developed a flow-extension single-molecule assay capable of examining the lengthening of ssDNA induced by the binding of multiple MutL proteins (Fig. 5A–C). One end of a 3′-biotin 5.3 kb ssDNA was linked to a PEG-biotin surface via streptavidin. The opposite end containing digoxigenin was attached to a 2.8 µm diameter super-paramagnetic bead coated with anti-digoxigenin antibody (Fig. 5A). A force was applied to the bead in the flow chamber, with the net force given by a magnetic force perpendicular to the surface and a laminar flow parallel to the surface, that ultimately results in stretching of the ssDNA (Fig. 5A; Materials and Methods). MutL was injected into the flow chamber with a constant flow rate corresponding to a net force of 2.5 pN. The change in the length of individual ssDNA molecules was monitored by the position change of the bead linked to the ssDNA.

Injection of MutL (200 nM) resulted in the gradual length extension of the ssDNA to 1 µm by ∼100 s (Fig. 5D). Washing free MutL protein from the flow chamber at 400 s resulted in a slow shortening of the extended ssDNA (Fig. 5D). These results demonstrate the association and dissociation of MutL from the ssDNA (Fig. 5B and 5C). We did not observe any change in the length of the ssDNA in the absence of ATP as we expected from the smFRET experiment. A length change to nearly 1 µm strongly suggests that multiple MutL proteins are binding to the ssDNA. To confirm this notion, we examined the MutL concentration dependence of ssDNA extension (Fig. 5D and 5E). We found that the maximum ssDNA extension was dependent on MutL concentration (S_0.5_ = 24 nM) that saturated at a length of approximately 1 µm, which is equivalent to 30% of the length of the fully stretched ssDNA. The rate of extension was observed to be linearly proportional to the concentration of MutL (Fig. 5F). These results parallel the observation that the kinetic rate of association (*k_on_*) is proportional to MutL concentration (Fig. 5B), and suggest that ssDNA length extension is controlled by the rate of MutL association.

We performed MutL-ssDNA extension studies in a range of ionic conditions (Fig. 5G). We used 200 nM of MutL that appeared saturating for ssDNA binding. Between 0 and 25 mM of NaCl the ssDNA extension was maximized (946±77 nm at 25 mM, mean ± s.e.m.; Fig. 5H). At 100 mM NaCl the extension of ssDNA decreased to 297±17 nm (mean ± s.e.m.) and at salt concentrations above 100 mM (120 mM and 150 mM) no significant change in length of ssDNA was observed (Fig. 5G). These observations are consistent with the smFRET studies and suggest that multiple MutL proteins do not stabilize ssDNA binding, and that polymerization of MutL on ssDNA is unlikely to occur at physiological salt conditions [12].

## Discussion

We have developed two single-molecule assays and demonstrated ATP-dependent MutL-ssDNA binding at ionic strength below 100 mM. Our work represents the first single-molecule analysis of MutL-ssDNA interactions in real time. Very recently, Gorman *et al.* reported that human MutL homolog (Mlh1/Pms1) moves on dsDNA with a mean diffusion coefficient, 0.143±0.29 µm^2^/s, at 150 mM NaCl [21]. However *E. coli* MutL bound to ssDNA does not seem to diffuse along ssDNA, which is supported by our observations of the identical off-rate between the end-free and the end-blocked ssDNA.

In our smFRET assay, Cy3 binding by MutL might cause a low FRET due to the enhancement of Cy3 intensity. It is known that binding or proximity to a single fluorophore by unlabeled proteins can induce the intensity enhancement of the fluorophore [22], [23], [24]. To test this, we investigated the Cy3 intensity in the presence of MutL with the 33 nt ssDNA substrate eliminating Cy5 at the junction. We found no intensity changes. These results confirm that the FRET change we observed occurred by the distance change between a donor and an acceptor owing to ssDNA binding by MutL.

The results presented here are consistent with previous studies that have demonstrated ssDNA binding by MutL [4], [5], [6], [9]. At low ionic strength, MutL-ssDNA binding is controlled by a protein concentration dependent first-order on-rate (*k_on_*). Increasing ionic strength increases the off-rate (*k_off_*) more than 3-fold; presumably escalating it such that binding is not observed above 100 mM. These results suggest a salt masking effect where stable MutL-ssDNA contact(s) are either eliminated or substantially reduced by increasing ionic strength [20]. We conclude that there are unlikely to be stable or long-lived interactions between MutL and ssDNA at physiological salt as suggested by Acharya et al. [13].

We found that MutL can stretch ssDNA, which allows us to observe an individual MutL binding to ssDNA in the smFRET assay. In addition, MutL polymerization on a long ssDNA (5.3 kb) could also be explored using the flow-extension assay. However the length change of dsDNA was not observed in the presence of dsDNA in our single-molecule assays. The extension mechanism associated with MutL-ssDNA binding is unclear. Interestingly, other biochemical studies showed that MutL and MutL(D58A) bound to 92/93 bp partial duplex DNA in the absence of ATP [4], [5]. Yet, MutL only bound to ssDNA in the presence of ATP [4], [5]. Taken together, we speculate that the FRET changes observed in our studies are likely to result from DNA interaction(s) by two distinct sites within the MutL homodimer that causes stretching of the random coiled ssDNA tail when the N-terminal domains form an ATP-induced dimeric structure.

A binding association that is eliminated by ionic strength may be indicative of alternative MutL-ssDNA interaction(s) or alternate interactions in the presence of additional MMR proteins. Genetic studies have demonstrated that the MutL(R266), residue implicated in ssDNA binding, is required in MMR [5]. The MutL(R266) residue is located in a cleft formed by the ATP-binding controlled homodimerization N-terminal LN40 domain. Moreover, the cleft is located in a hole formed by the connection of the C-terminal LC20 homodimer interaction domains via a flexible linker. Thus, the ATP-dependent dimerization of the N-terminal LN40 domain would appear to form a cavity containing the MutL(R266) residue much like the cavity formed by MSH protein clamps on mismatched DNA [25], [26], [27]. Interestingly, MLH/PMS ssDNA-dependent ATPase activity has only been performed at or below 90 mM NaCl [5], [10], [12], potentially suggesting an inverse correlation with salt concentration. We regard it possible that such a dynamic ATP-dependent conformational transition may allow transient interaction(s) during the excision reaction that ultimately positions a displaced ssDNA strand in the MutL cavity [13]. Alternatively, interactions between downstream effectors such as UvrD or one of four exonucleases required for MMR may enhance MutL-ssDNA binding by altering local ionic conditions. However, it appears clear that polymerization of MutL along ssDNA as a mechanism in MMR is unlikely to occur under physiologically relevant conditions [12].

## Materials and Methods

### Protein purification of *E. coli wild type* MutL, MutL(D58A), MutL(R266E)

Cloned hexahistidine tagged *E. coli* wtMutL and mutant MutL(D58A, R266E) in a pET15b-TEV were overexpressed from the *E. coli* strain BL21(DE3). Harvested cells were resuspended in 100 mL of lysis buffer (20 mM Tris-HCl, pH 8.0, and 0.5 mM β-mercaptoethanol). Cells were lysed by adding 1 mg/mL lysozyme, which was followed by sonication. Lysates were centrifuged and the supernatant was applied to a 5 mL HisTrap™HP (GE Healthcare) that was pre-equilibrated with a binding buffer (20 mM Tris-HCl, pH 8.0, 0.5 mM β-mercaptoethanol, and 500 mM NaCl). After the column was washed with a binding buffer that contains 15 mM imidazole, proteins were eluted with the binding buffer that contains 300 mM imidazole. The wtMutL and mutant MutL from the Ni-column were directly injected into a desalting G-25 column that was pre-equilibrated with a desalting buffer (20 mM Tris-HCl, pH 8.0, 0.5 mM β-mercaptoethanol, and 125 mM NaCl). Then, the proteins were applied to a MonoQ HR 10/10 column (GE Healthcare) that was pre-equilibrated with buffer A (20 mM Tris-HCl, pH 8.0, 1 mM EDTA and 1 mM DTT, 5 mM MgCl_2_) and eluted with a linear gradient from 25% to 100% of buffer B (20 mM Tris-HCl, pH 8.0, 1 mM EDTA, 1 mM DTT, 5 mM MgCl_2_ and 500 mM NaCl). The wtMutL and mutant MutL were eluted at 190–220 mM NaCl. The dimeric proteins were purified with a Superdex 200 HR 10/30 column (GE Healthcare) with buffer C (20 mM Tris-HCl, pH 8.0, 0.5 mM β-mercaptoethanol, and 125 mM NaCl) to obtain greater than 95% purity. The concentration of the proteins was kept in less than 0.5 mg/ml to avoid self-aggregation. More detailed information for strains, plasmid, and protein purification was described in Ref. [5], [9].

### Single-molecule FRET

#### Experiment setup

To construct partial duplex DNA substrates, PAGE or HPLC-purified oligodeoxynucleotides that were modified with biotin, Cy3, Cy5, and digoxigenin were purchased from IDT (Coralville, USA): Cy3-dT33 oligo (Cy3-5′-dT_33_CGA CGG CAG CGA GGC-3′), Dig-dT(33+11) oligo (dig-5′-dT_11_-Cy3-dT_33_CGA CGG CAG CGA GGC-3′), Dig-dT(15+3) oligo (dig-5′-dT_3_-Cy3-dT_15_CGA CGG CAG CGA GGC-3′), and biotin-Cy5 oligo (Biotin-5′-GCC TCG CTG CCG TCG-3′-Cy5). Partial duplex substrates that consist of 15 bp duplex with 33 nt, 15 nt, 44 nt (dig), and 18 nt (dig) 5′-overhang were prepared by annealing a pair of biotin-Cy5 and Cy3 oligos (Cy3-dT33 oligo, Cy3-dT15 oligo, Dig-dT(33+11) oligo, Dig-dT(15+3) oligo) at a molar ratio of 1∶1.1 in the annealing buffer (10 mM Tris-HCl, pH 8.0, 100 mM NaCl, 1 mM EDTA) for a final concentration of 4 µM, respectively. The solution that contains the oligos was incubated at 95°C for 5 min and was then slowly cooled down to room temperature over 3 h. The annealed DNA substrates were stored at 4°C.

Quartz glass was functionalized with PEG-biotin and PEG (1∶40 in a mass ratio, Laysan Bio), while cover glass was functionalized only with PEG to minimize the nonspecific binding of the DNA substrates or proteins [28]. Streptavidin in PBS (4 µM in 125 µl, Sigma) was spread on the surface of the quartz glass and incubated for 30 min. The quartz glass was washed with double distilled water and dried with a nitrogen gas jet. A flow chamber with a channel of 25 mm×3 mm×0.1 mm that was generated using a double sticky tape (Biolabs) was constructed with the streptavidin-coated quartz glass and PEG-only-functionalized cover glass. To immobilize the DNA substrates, 10 pM DNA in the blocking buffer (20 mM Tris-HCl, pH 7.5, 2 mM EDTA, 50 mM NaCl, 0.0025% Tween 20 (v/v), 0.1 mg/ml BSA) was incubated in the flow chamber for 5 min. Free DNA was removed by extensive washing with blocking buffer. Reaction buffer consisted of 20 mM Tris-HCl, pH 7.5, 25–150 mM NaCl, 0.1 mM EDTA, 3 mM MgCl_2_, 0.5 mM ATP, and 1 mM DTT. Proteins in the reaction buffer were injected into the chamber to measure binding to DNA substrate. To increase the photostability of the dyes, 2 mM of trolox, 0.8% (w/v) of D-glucose, 165 U/ml of glucose oxidase, and 2,170 U/ml of catalase were added as an oxygen-scavenging system in the reaction buffer [29].

Emission signals from a donor excited with a 532 nm DPSS laser (Cobalt, 100 mW) and an acceptor excited by energy transfer were collected and recorded using EM-CCD (Andor iXon^EM+^897), with lab-developed imaging software and a 50 ms time resolution. To image the fluorescent signals, we used a wide-field total internal reflection fluorescence (TIRF) microscope with water-immersion objective (60×, N.A. = 1.2, Olympus), for which the total internal reflection of an incident beam was induced by a prism.

#### Data analysis

The data were analyzed using IDL and MATLAB scriptures obtained from Ha group at the University of Illinois (http://bio.physics.illinois.edu). After the corrections of the donor (*I_D_*) and the acceptor (*I_A_*) intensities for cross-talk between their channels as well as for the background, FRET efficiencies were calculated as the ratio of *I_A_* to *I_D_+I_A_*. Each of the single traces was processed using hidden Markov modeling (HMM) with maximum evidence to identify multiple states without personal prejudice [30]. The software is available at http://vbfret.sourceforge.net.

Dwell time at the states determined by HMM analysis was used to calculate the kinetic parameters of an on-rate (*k_on_*) and off- rate (*k_off_*) in the following reaction.
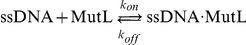
Histogram of the dwell time (binding time : *τ_on_*, unbinding time : *τ_off_*) at each state was fitted by an exponential function of exp(*−k_off_·t*) or exp(*−k_on_·t*) for a single rate reaction, where *k_off_* = 1/*τ_on_* as an off-rate and *k_on_* = 1/*τ_off_* as an on-rate.

### Flow-extension assay

For the flow-extension experiments, we constructed a 5.3 kb ssDNA as follows: 1) λ phage DNA (New England Biolabs) was digested with BsrGI (New England Biolabs); 2) a resulting 5,208 bp left-arm fragment that contains a 4 nt BsrGI 5′-overhang and the λ DNA left cohesive-end was isolated from a 7% agarose gel (QIAquick Gel Extraction Kit, QIAGEN); 3) the 12 nt λ-tail was annealed and ligated with a 3′biotin oligo (5′-AGG TCG CCG CCC AGT TAC AGA TTT ATG GTG ACG ATA CAA ACT ATA GAG TGA (dT)_43_-3′-biotin); 4) the 4 nt BsrGI-tail was annealed and ligated with a 5′-digoxigenin (Dig) oligo (Dig-5′-(dT)_12_ TGA TGA ATT CTA ATG-3′) and a complementary linker oligo (5′-GTA CCA TTA GAA TTC ATC A-3′); and 5) the 5,333 nt ssDNA bearing both 3′-biotin and 5′-digoxigenin was obtained by heating the constructed dsDNA in a 2 mM NaOH solution at 99°C for 5 min, and subsequently quenching it in a 4°C blocking buffer to prevent them from reannealing.

The cover glass was functionalized with PEG-biotin and PEG (with a mass ratio of 1∶100, Laysan Bio). A flow chamber was developed similar to that for the FRET studies. The chamber was placed on the stage of an inverted optical microscope (IX51, Olympus). The 5.3 kb ssDNA (0.5 pM) in the blocking buffer was incubated in the flow chamber for 10 min and unattached DNA was removed by extensive washing as described for the FRET studies. A super-paramagnetic bead (2.8 µm in diameter, Invitrogen) that was coated with anti-digoxigenin Fab (Roche) was linked to Dig-end of the ssDNA by flowing the beads into the flow chamber in the blocking buffer. Prior to the addition of MutL, the free beads were stringently removed by extensive washing [17].

A drag force parallel to the bottom surface was applied to a tethered bead from a laminar flow produced by a syringe pump (Harvard apparatus). A magnetic force generated by a rare earth magnet (NdFeB) upward from the surface was also applied to avoid nonspecific interaction(s) between the bead and the surface. The hydrodynamic and the magnetic forces were calculated by measuring the mean-square displacement 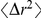 in the transverse direction to the stretching force, for which the bead position was measured at 50 Hz with 100× objective in a bright field optical microscope. The force (*F*) was determined as 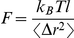, where *k_B_* is the Boltzmann constant, *T* is the absolute temperature, and *l* is the length of DNA [31]. Our studies were performed under 2.5±0.4 pN, which results from a vector summation of the magnetic force (1.1±0.4 pN) and the hydrodynamics force (2.2±0.2 pN) by a laminar flow. The error of the force represents s.e.m.

The beads were imaged through a 10× objective (N.A. = 0.40, Olympus). We observed more than 150 beads in a field of view. The diffraction patterns of the beads were recorded with a high-resolution CCD (RETIGA 2000R, QImaging) using MetaVue (Molecular Devices) imaging software. The bead positions that were recorded with a 500 ms time resolution were determined using 2D Gaussian fitting with a 10 nm accuracy [32]. The data were analyzed by DiaTrack 3.0 (Semasopht) and OriginPro 8 (OriginLab).

## Supporting Information

Figure S1 **Histogram of FRET values from populations of single molecules normalized to the peak count.** FRET efficiency in the presence of D58A MutL is 0.43±0.18 (mean ± s.d.) that is very similar to that (0.41±0.15) in the absence of MutL. (TIF)
